# Similar digit-based working memory in deaf signers and hearing non-signers despite digit span differences

**DOI:** 10.3389/fpsyg.2013.00942

**Published:** 2013-12-16

**Authors:** Josefine Andin, Eleni Orfanidou, Velia Cardin, Emil Holmer, Cheryl M. Capek, Bencie Woll, Jerker Rönnberg, Mary Rudner

**Affiliations:** ^1^Linnaeus Centre HEAD, Swedish Institute for Disability Research, Department of Behavioural Sciences and Learning, Linköping UniverstiyLinköping, Sweden; ^2^Department of Psychology, University of CreteRethymnon, Greece; ^3^Deafness Cognition and Language Research Centre, Department of Cognitive Perceptual and Brain Sciences, University College LondonLondon, UK; ^4^School of Psychological Sciences, University of ManchesterManchester, UK

**Keywords:** deaf signers, working memory, short-term memory, phonological similarity, cross-culture

## Abstract

Similar working memory (WM) for lexical items has been demonstrated for signers and non-signers while short-term memory (STM) is regularly poorer in deaf than hearing individuals. In the present study, we investigated digit-based WM and STM in Swedish and British deaf signers and hearing non-signers. To maintain good experimental control we used printed stimuli throughout and held response mode constant across groups. We showed that deaf signers have similar digit-based WM performance, despite shorter digit spans, compared to well-matched hearing non-signers. We found no difference between signers and non-signers on STM span for letters chosen to minimize phonological similarity or in the effects of recall direction. This set of findings indicates that similar WM for signers and non-signers can be generalized from lexical items to digits and suggests that poorer STM in deaf signers compared to hearing non-signers may be due to differences in phonological similarity across the language modalities of sign and speech.

## INTRODUCTION

Working memory (WM) is the limited capacity available for maintaining and processing information online and is thus vital for communication (Baddeley, 2003). The ability to maintain and process lexical items in WM has been shown to be equal, irrespective of whether those items are signs or words (Boutla et al., 2004; Rudner et al., 2007, 2013). However, short-term memory (STM) capacity (i.e., maintenance only) is generally lower for signs than words (Marschark and Mayer, 1998; Boutla et al., 2004; Rönnberg et al., 2004; Geraci et al., 2008). In an attempt to settle the discrepancies in capacity estimates, the aim of the present study was to investigate STM for digits and letters as well as WM for digits in deaf signers and hearing non-signers by making direct comparisons between well-matched groups. Text-based presentation was used throughout and influence of response mode was investigated.

Sign languages are natural, complex visual languages of deaf communities (for review, see Emmorey, 2002). They can be described using the same terminology as speech-based languages, such as phonology, morphology, syntax, and prosody (Klima and Bellugi, 1979; Sandler and Lillo-Martin, 2006). Although sign languages are not representations of either spoken or written language, many, although not all, sign languages make use of manual alphabets (fingerspelling) to represent letters and orthography when producing, e.g., place names or proper names (Brentari, 1998). There are many different one- and two-handed manual alphabets (Carmel, 1982), including the Swedish manual alphabet, which is one-handed, and the British manual alphabet, which is two-handed. Both these alphabets are used productively to fill lexical gaps (Bergman and Wikström, 1981; Sutton-Spence and Woll, 1999). For spoken language, phonology is concerned with the combination of sounds to form utterances, while for signed language phonology refers to how sublexical components of signs are put together with respect to handshape, position (including orientation), and movement (Sandler and Lillo-Martin, 2006). Signs that share one or more realizations of these features are considered to be phonologically similar (Klima and Bellugi, 1976; Sandler and Lillo-Martin, 2006).

Traditionally, WM and STM are measured using span tests. WM can be measured by, e.g., reading span, listening span, or operation span, where operation span has been shown to load most strongly on overall WM capacity (Unsworth and Engle, 2007). The operation span task (Turner and Engle, 1989) is a WM task in which arithmetic operations are performed on quantities represented by digits and the results of these operations are serially maintained in memory for subsequent recall while new operations are performed. Thus, the operation span task relies on manipulation of abstract representations but without necessarily calling on linguistic structure. While STM ability, measured by simple spans, for deaf signers and hearing non-signers has been investigated extensively, WM, measured by complex spans, has only been investigated for these groups in two studies (Boutla et al., 2004; Alamargot et al., 2007). In both studies a production span task was used and no differences between groups were found for either adults (Boutla et al., 2004) or children (Alamargot et al., 2007). Studies using other tests aimed to investigate WM capacity have also concluded that there are no general differences between deaf signers and hearing non-signers in WM (Rudner et al., 2010, 2013). All of the above mentioned studies have however, either used linguistic stimuli, such as signs and words, or easily nameable pictures. In the present study we will investigate if similar WM capacity for deaf signers and hearing non-signers can be generalized to digit-based WM.

Short-term memory is frequently assessed, in both research and neuropsychological testing, by the digit span test. A substantial body of literature has shown that deaf signers perform at a lower level on this test than hearing non-signers, even when the test is administered in their native sign language, and despite equal performance on other cognitive tasks (Pintner and Paterson, 1917; Wilson et al., 1997; Bavelier et al., 2008). This phenomenon is not related to deafness *per se*, but to the use of sign language, since this difference is also found in hearing signers (Boutla et al., 2004). There are several potential underlying mechanisms explaining the differences in STM between the language modalities of sign and speech, among them the phonological similarity effect (Baddeley et al., 1975; Wilson and Emmorey, 1997a) and the temporal order effect (Wilson and Emmorey, 1997b; Wilson et al., 1997).

**FIGURE 1:**
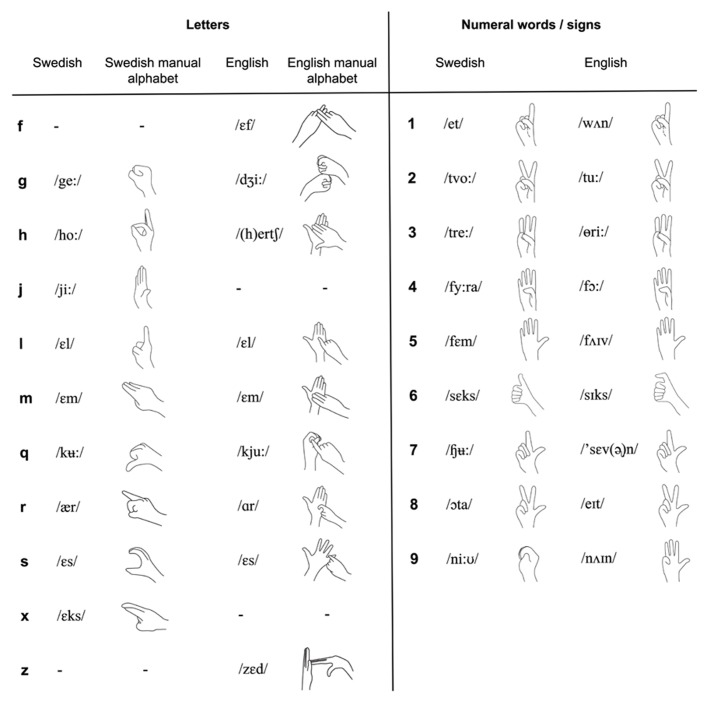
**Phonology of the nine fingerspelled letters of the Swedish manual alphabet in Experiment 1, the nine fingerspelled letters of the British manual alphabet used in Experiment 2 and the nine manual numerals according to the Swedish and British systems for the digits 1–9 and numeral words and letters as expressed by the international phonetic alphabet for pronunciation in Swedish and English.** Note that the illustrations for BSL manual numerals represent the traditional manual numerals used in London, from which most of the BSL users in the present study originated, but there is extensive variation, in particular in the numbers 6–9.

The phonological similarity effect arises because items with similar sublexical structure give rise to similar traces in the phonological loop, resulting in confusability (Baddeley et al., 1975). This applies to words that sound similar, such as words that rhyme with each other and to signs with similar formational properties such as same handshape (Wilson and Emmorey, 1997a). Confusability of memory traces means that serial recall is less accurate for items that are phonologically similar (Baddeley et al., 1975; Wilson and Emmorey, 1997a). In most spoken languages, including English and Swedish, digit names are phonologically dissimilar, whereas in many sign languages, the numeral signs representing digits are phonologically similar as they share the same position, orientation, and movement and differ only in handshape, and then only minimally (see **Figure 1**). Thus, a higher digit span size for speakers than for signers (Pintner and Paterson, 1917; Bavelier et al., 2008), may at least partially be due to the phonological similarity of manual numerals. On the basis of this it has been suggested that letter span would be a better instrument to use when STM is compared between signers and speakers (Boutla et al., 2004). Indeed, Wilson and Emmorey (2006a) reported similar STM spans for deaf signers and hearing non-signers for a single set of letters (fingerspelled for the deaf signers and spoken for the hearing non-signers), matched for articulatory duration and phonological similarity in American sign language (ASL) and English, suggesting that STM for ASL and English do not differ in underlying capacity. However, this conclusion was contested by Bavelier et al. (2006), who provided evidence of lower STM span for signers than speakers with two sets of letters whose names were duration-matched across language modality. Thus, evidence for the role of phonological similarity in span differences between languages is inconclusive. In this study we use both digit span (phonologically similar in sign language and dissimilar in spoken language) and letter span (dissimilar in both languages) to pinpoint the phonological involvement in STM.

The temporal order effect arises because the auditory system is efficient in retaining temporal order, in contrast to the visual system (Smyth et al., 2005). In memory tasks, forward recall preference has been reported for hearing individuals (Rönnberg et al., 1980; Li and Lewandowsky, 1995; Rosen and Engle, 1997). Studies of deaf signers, however, report reduced forward preference (Bavelier et al., 2008) and backward or no temporal preference (O’Connor and Hermelin, 1973), although the more deaf individuals rely on a speech-based code, the better they are at maintaining temporal order (Hanson, 1982). This implies that there might be a preference for temporal encoding in speech, with signers using spatial encoding to a larger extent, especially when they do not rely on speech encoding. In support of this view, several studies have demonstrated that hearing individuals prefer to recall information in the same temporal order as presented, while deaf individuals preferred spatial recall (O’Connor and Hermelin, 1973; Rönnberg and Nilsson, 1987). When temporal processing demands are relaxed, memory differences between signed and spoken language disappear (Hanson, 1982; Boutla et al., 2004; Bavelier et al., 2008; Rudner and Rönnberg, 2008; Rudner et al., 2010). In the present study we examine the temporal order effect by administering both forward and backward span tests.

Much of the recent work investigating STM for sign language has used signed stimuli with signed response for signing participants who are either deaf or hearing and auditory stimuli with spoken response for hearing speakers (Wilson and Emmorey, 1998, 2006a; Boutla et al., 2004; Hall and Bavelier, 2011). The main advantage of this approach is that it allows individuals to perform the tasks in their own language with optimized stimulus presentation and recall mode for both language modalities. Another advantage is that it has made it possible to test hearing users of sign languages in both spoken language and sign language (Hall and Bavelier, 2011), providing experimental control in a within-groups design. However, this design has the drawback of introducing confounds in the experimental situation when stimulus and response modes differ between language modalities (Koo et al., 2008). Because auditory memory traces persist longer than visual memory traces (Sperling, 1960; Darwin et al., 1972), hearing individuals presented with auditory stimuli can take advantage of a more capacious buffer that reduces the load of the rehearsal process to a greater extent than can deaf individuals presented with visual stimuli (Cowan, 2000). In the present study we used printed characters as stimuli and written response for all participants. In one sense this returns to an older tradition which expected deaf individuals to perform memory tasks in speech-based languages which may not have been their preferred mode of communication (Pintner and Paterson, 1917; Ross, 1969; Conrad, 1970; Locke and Locke, 1971; Wallace and Corballis, 1973). However, our assumption was that all participants would recode the characters as the appropriate lexical labels in their preferred language modality during memory encoding and reverse this process at recall. To ensure best possible compliance to our assumption, we recruited participants whose language experience was orthogonal to each other: deaf signers and hearing non-signers. Both the Swedish deaf signers in Experiment 1 and the British deaf signers in Experiment 2 had learned sign language at an early age and used it as their preferred language in everyday communication. The hearing non-signers in these experiments had no knowledge of sign language.

In sum, the aim of the present study was to investigate digit-based WM and STM in deaf signers and hearing non-signers. The first experiment included deaf signers (SDS) who use Swedish sign language (SSL) and hearing Swedish speakers (SHN) with no knowledge of sign language. We tested WM using the operation span task and STM with a 2 × 2 × 2 experimental set-up which included digit and letter span tasks with forward and backward recall. Text-based presentation on a computer screen was used throughout and key press response mode was kept constant across groups. The second experiment repeated the first but with handwritten responses and in a different cultural and linguistic setting with deaf signers (BDS) who are users of British sign language (BSL) and hearing speakers of British English (BHN) with no knowledge of sign language. The third experiment examined the effect of mode of recall: key press or handwritten response on STM in a within-groups design with hearing Swedish speakers.

We predicted similar WM, measured by operation span, for deaf signers and hearing non-signers in line with previous work showing similar production spans (Boutla et al., 2004). We expected that differences in STM relating to temporal processing demands and language modality-specific phonological similarity would reveal causes of the shorter STM spans generally found in deaf signers compared to hearing non-signers. In particular, we expected shorter digit but not letter spans for deaf signers than hearing non-signers and shorter backward than forward spans for hearing non-signers but not deaf signers.

## EXPERIMENT 1

### MATERIALS AND METHODS

#### Participants

Eighteen Swedish deaf adults (14 women; mean age = 27.83, *SD* = 4.08) were recruited to the study. All participants were deaf from birth (17 participants) or before 6 months of age (one participant) and all were native or early signers who started to use SSL before the age of three. Four participants had deaf parents who signed with them from birth and one had a deaf older sibling and was therefore signed with from birth. All deaf participants born after 1976 went to deaf schools using the 1983 Swedish national curriculum with education mainly in SSL (Svartholm, 2010). One deaf participant born before 1976 started to use SSL at the age of two and worked as an accredited sign language teacher. Thus, we consider the SSL proficiency of this individual to be on a par with that of the rest of the deaf participants. All deaf participants considered SSL to be their first language; they used SSL in all one-to-one communication, and were reluctant to use oral communication and lip reading. However, they were all capable of communicating in written Swedish.

Eighteen adult hearing, native Swedish speakers (14 women; mean age = 28.17, *SD* = 5.52) were also included in the study. The Swedish speakers were unfamiliar with sign language. The participants in the spoken language group were recruited to match the signing group on age, *t*_(34)_ = 0.21, *p* = 0.84, and education. The two groups were further matched on non-verbal IQ (Raven’s standard progressive matrices, *t*-scores; SDS *M* = 50.94 *SD* = 7.93, SHN *M* = 54.94 *SD* = 7.02; *t*_(34)_ = 1.60, *p* = 0.12). All participants had completed mandatory Swedish education, which at the time meant 9 years for SHN and 10 years for SDS, and Swedish high school (3–4 years). Six of the SHN and six of the SDS had a university degree or equivalent education (e.g., as sign language teachers). Thus, we used a participant base with no statistically significant differences between the groups regarding sex, age, non-verbal IQ, and education. We consider this careful matching to be of great importance and such matching differentiates this study from previous studies where groups have been less well-matched (typically only on gender and age).

The group manipulation in this study is language modality. The groups also differ in that one group consists of deaf individuals and the other of hearing individuals. However, we consider language modality to be the differentiator in serial recall tasks as it has been shown that increased reliance on speech-based code leads to smaller differences between hearing and deaf individuals (Hanson, 1982).

The study was approved by the regional ethics committee in Linköping (Dnr 190/05). Written informed consent was given by all participants. The participants were compensated for their travel expenses and offered a small gift of nominal value after completing the study.

#### Stimuli

The test battery used in this study comprised five span tests, which were performed in the following order for all participants: digit span forward and backward, letter span forward and backward, and operation span. All stimuli were presented as printed characters at the center of a computer screen keeping mode of presentation constant across conditions. The tests were performed individually. Verbatim translation of test instructions from Swedish to SSL for deaf participants was performed by professional sign language interpreters, and participants could ask questions. In the same session, before the span tests, tests of phonology and arithmetic were administered to all participants as part of a larger study.

#### Working memory test

The WM measure was the dual-task operation span test based on Turner and Engle (1989). Forty-two equations each consisting of two operations were used as stimuli. The first operation in the string was either multiplication or division and the second operation was either addition or subtraction (e.g., 3 × 2 + 1 = 7). Single digit numbers 1–9 were used throughout the test. Twelve sequences, half true and half false, were created (2–5 strings progressively increasing in size, three sequences of each length). The task of the subjects was to report, by key press, if the stated answer was correct or not. After each sequence the subjects were instructed to recall all the stated answers in the same order as presented. Data were collected from both the recall and the manipulation phase. Generally, deaf individuals have been shown to have poorer mathematical ability than hearing peers (Traxler, 2000), which could affect performance on the operation span task. However, the careful matching of performance on Raven’s matrices and education was an attempt to handle this potential problem. To further ensure that all participants had basic mathematical knowledge, all subjects were tested on a simple digit task where they were asked about numerical order. No language modality differences were seen on this task, *t*_(27)_ = 1.46, *p* = 0.154.

#### Short-term memory tests

For the digit span tests, digits from 1 to 9 were chosen to create random sequences of 2–9 items. For the letter span tests, sequences of nine letters that represent consonants, chosen to minimize phonological similarity in both SSL and Swedish, were created (G, H, J, L, M, Q, R, S, and X; **Figure 1**). Phonological similarity was minimized by avoiding letter names that either rhymed with each other in Swedish or shared the same handshape in Swedish sign alphabet. Sixteen meaningless sequences were created randomly (2–9 items, two sequences of each length). All participants were first exposed to two trials consisting of a sequence of two items. Then, the sequence length increased progressively, by one digit/letter at a time.

#### Procedure

All stimuli were presented visually using Presentation 14.2 software on a PC. Stimulus materials were presented in Times New Roman with a font size of 90. For the STM tests each digit or letter was presented for 1 s on a computer screen. For the WM task each equation appeared on the screen for 5 s. At the end of the sequence the participants were asked to enter the digits or the letters in presentation order for forward spans and operation span, and in the reverse order for backward spans, by using the number pad of the computer keyboard. Instead of using the full keyboard for letters the participants used a number pad where each digit key had been covered with a label displaying a letter (7 = G, 8 = H, 9 = J, 4 = L, 5 = M, 6 = Q, 1 = R, 2 = S, and 3 = X). The letters were arranged on the number pad in alphabetical order from top left to bottom right. It was not possible to use a mobile phone analogy as G and H, for example, both map onto number 4. There is no reason to believe that the adopted mapping would cause more interference for one group than the other. It could be argued that the individual letters are more difficult to locate than the individual digits as the letters are represented by an unfamiliar set of keys. However, theoretically the benefits of this set-up outweigh the drawbacks. With oral recall, handwritten recall or recall using the entire alphabetic keyboard, it is possible for the participants to erroneously include letters other than the nine chosen, producing a bias compared to the digits which are limited in a more natural way. Even if the alphabetic keyboard were restricted by covering or removing irrelevant keys, the visuospatial demands would have been different to those of the number pad. In our experiment, the set of possible letter responses was constrained to the nine letters included in the experiment, equalizing response demands between letters and digits and between groups. At the same time the possible effect of keyboard skills was reduced. Further, key press response provides the opportunity to compute exact response times for each given answer.

The participants were asked to respond to all sequences to the best of their ability and to include all items they could remember even if they did not remember the exact serial order. During recall the participants’ letter and digit responses did not appear on the screen, instead the screen remained black with only the cue for recall visible (square brackets). This arrangement was chosen to be analogous to oral recall where no visual feedback is available.

#### Scoring

Span size was scored as the length of the complete sequence at which the participant recalled at least one of the recall attempts in correct serial order (Unsworth and Engle, 2007). The maximum possible span size was nine in the STM tests and five in the WM test. For the WM task, the proportion correct scoring (PCS) procedure was also applied (Unsworth and Engle, 2007). This procedure is deemed appropriate for complex spans and represents the proportion of items that are recalled in correct serial position (maximum score 42). For the WM task a measure of the math process component was also obtained. The math process component was defined as the number of correct answers (maximum 42).

#### Statistics

The WM task was analyzed by a *t*-test with language modality as between subject factor. The design for the STM tasks was a 2 × 2 × 2 (language modality [SDS, SHN] × character [digits, letters] × direction of recall [forward, backward]) design, which was investigated by analysis of variance (ANOVA). The between subject factor was language modality (SDS and SHN) and the within subject factors were direction of recall (forward and backward) and character (digit and letter). Planned comparisons of shorter digit span but similar letter span for deaf signers compared to hearing non-signers and shorter backward than forward span in hearing non-signers were conducted by a series of *t*-tests. Other simple main effects were only analyzed if the corresponding interaction effect was significant at *p* < 0.05. Data analysis was performed using IBM SPSS, version 21.

### RESULTS

There was no significant difference between groups on the WM task measured by either span size, *t*_(33)_ = 0.97, *p* = 0.34, *r* = 0.17, or PCS, *t*_(33)_ = 1.25, *p* = 0.22, *r* = 0.21 (**Table 1**). We also analyzed the responses given during the manipulation phase of this task and found no significant differences between language modalities, *t*_(33)_ = 1.87, *p* = 0.29, *r* = 0.31 (SHN: *M* = 35.61, *SD* = 5.50, SDS: *M* = 33.06, *SD* = 8.20). This ruled out the possibility of difference in strategies between groups.

**Table 1:** Span size and proportion correct scores (PCS) for operation span performance in experiments 1 (Swedish participants) and 2 (British participants).

		Span size		PCS	
		*M*	*SD*	*M*	*SD*
Swedish	deaf signers	3.47	1.62	0.66	0.23
	hearing non-signers	3.94	1.26	0.75	0.20
British	deaf signers	4.50	1.22	0.83	0.16
	hearing non-signers	4.30	1.39	0.82	0.20

**FIGURE 2:**
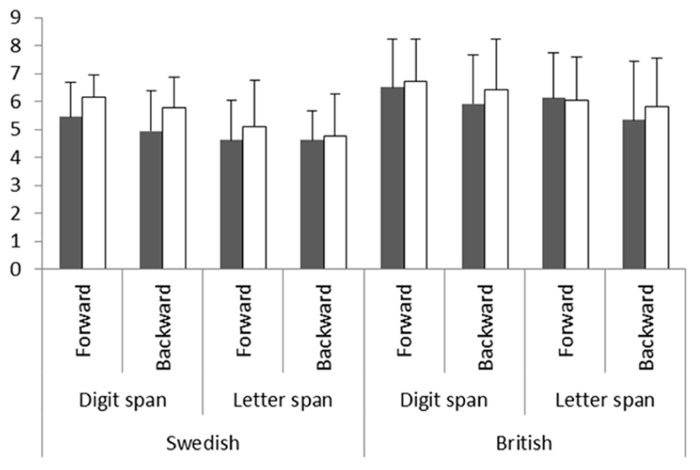
**Mean forward and backward digit and letter spans (and standard deviation) in Swedish and British deaf signers (grey bars) and hearing non-signers (white bars)**.

When we analyzed the STM experiment, we found that SHN tended to perform better than SDS, *F*_(1,34)_ = 3.35, *p* = 0.08, *partial* η^2^ = 0.09. There was a significant main effect of character *F*_(1,34)_ = 23.81, *p* < 0.001, *partial*η^2^ = 0.41 (**Figure 2**), demonstrating that digit span was longer than letter span. The interaction between group and character did not reach significance, *F*_(1,34)_ = 1.812, *p* = 0.187, *partial*η^2^ = 0.05. However, because we predicted that digit but not letter spans would be shorter for SDS than SHN, we investigated this and found that whereas SHN had significantly longer digit spans than SDS, *F*_(1,34)_ = 6.21, *p* = 0.02, *partial* η^2^ = 0.15, there was no difference in letter spans, *F*_(1,34)_ = 0.79, *p* = 0.38, *partial* η^2^ = 0.02. Surprisingly, and in contrast to previous literature, there was no significant main effect of recall direction, *F*_(1,34)_ = 2.66, *p* = 0.11, *partial* η^2^= 0.07. The hypothesis that hearing non-signers but not deaf signers would have shorter backward than forward spans was tested and we found no effect of direction for either group (SDS: *F*_(1,17)_ = 0.68, *p* = 0.42, *partial*η^2^ = 0.04, SHN: *F*_(1,17)_ = 2.68, *p* = 0.12, *partial* η^2^ = 0.14). All other interactions showed *p* > 0.45.

### DISCUSSION

As predicted, we found no difference in performance between groups on the operation span task, which is a digit-based WM task. This extends previous results showing similar WM capacity for sign and speech on a production span task with lexical material (Boutla et al., 2004). Also as predicted, we found poorer digit but not letter span for signers compared to speakers. This indicates that the formational similarity of Swedish signed numerals generates a phonological similarity effect that depresses STM performance (Wilson and Emmorey, 1997a). However, this finding should be interpreted with caution given the fact that the language modality by character interaction was not statistically significant. Despite the fact that digit spans were reduced for SDS compared to SHN, they were still longer than letter spans. Longer digit than letter span in hearing signers has been described by Wilson and Emmorey (2006a), and may be due to the larger potential set size of letters compared to digits, and the unfamiliar mapping of letters to response keys in the experiment as well as articulation rate differences between digits and letters.

We found no significant effect of recall direction and no evidence to support our hypothesis that shorter backward than forward spans would only be found in the hearing group. Other recent work has failed to find temporal processing differences between deaf signers and hearing non-signers (Gozzi et al., 2011). However, this does not explain the absence of a recall direction effect. Immediate forward recall of spoken items is characterized by a substantial recency effect whereby those items encoded most recently are remembered best. The recency effect is reduced when items are written compared to auditory (Conrad and Hull, 1968; Beaman and Morton, 2000; Cowan et al., 2004; Harvey and Beaman, 2007) and a similar effect has been found for deaf signers when the comparison is made with signed stimuli (Shand and Klima, 1981). When the final items in a series are recalled first there is no difference between visual and auditory presentation, but if the final items are recalled last, higher performance is obtained if the presentation mode is auditory (Beaman and Morton, 2000; Cowan et al., 2004). Hence, derived predictions suggest better forward performance with auditory compared to visual presentation, and no difference for backward recall. This means that there is likely to be a smaller difference between forward and backward recall when presentation is visual. Further, key press response suppresses recency items more than handwritten response, leading to poorer recall, especially when presentation is visual (Penney and Blackwood, 1989). Hence, an effect of recall direction is more likely to be found with handwritten response. Thus, we used handwritten response in Experiment 2.

## EXPERIMENT 2

We wanted to investigate whether our findings relating to a phonological similarity effect for deaf signers in digit-based STM and no difference in performance between DS and HN on digit-based WM could be generalized to another cultural and linguistic setting. At the same time we wanted to investigate whether changing the response mode would give us an effect of recall direction and further whether that effect would differ between groups. Therefore, we repeated the experiment with equivalent groups but in Britain, using the same visual presentation as in Experiment 1, but instead of key press response, handwritten response was used. We hypothesized that handwritten response would result in significantly longer forward than backward spans in hearing non-signers but not in deaf signers. We also predicted that we would find similar WM performance across language modalities as well as shorter digit but not letter spans for deaf signers than hearing non-signers as we had found for Swedish participants in Experiment 1.

### MATERIALS AND METHODS

#### Participants

Twenty-four deaf adults (10 women; mean age = 38.58, *SD* = 12.15) were recruited to the study. All but two BDS were native signers having at least one deaf parent. One BDS with hearing parents started to learn BSL before the age of three and the other before the age of five.

Thirty adult hearing, native English speakers (20 women; mean age = 34.17, *SD* = 12.41) were also included in the study. The English speakers were unfamiliar with BSL. The participants in the speaking group were recruited to match the signing group on age, *t*_(52)_ = 1.31, *p* = 0.20. The two groups were further matched on non-verbal IQ (block design from Wechsler Abbreviated Scale of Intelligence, *t*-scores; BDS *M* = 61.83 *SD* = 6.68, BHN *M* = 60.13 *SD* = 7.64), *t*_(52)_ = 0.86, *p* = 0.40. All participants had completed mandatory British education, which at the time meant 10–12 years for both BHN and BDS.

The study was approved by the University College London Graduate School Ethics committee. Written informed consent was given by all participants. The participants were compensated for their time and travel expenses.

#### Experimental design

Stimuli, procedure, scoring, and statistics were the same as in Experiment 1 with the following exceptions: testing of the deaf participants was performed by a deaf experimenter who was a native signer of BSL, and testing of the hearing participants was performed by two hearing experimenters. Because of language differences, the letters J and X were changed to F and Z for the letter span task, in order to minimize phonological similarity in BSL fingerspelling and English letter names. Thus, the letters used in this experiment were F, G, H, L, M, Q, R, S, and Z (see **Figure 1**). Stimuli were presented visually using DMDX display software (Forster and Forster, 2003) on a PC. At the end of each sequence the participants were asked to use a pen to write down on a printed form the digits or letters in: presentation order, for forward spans and operation span; and in the reverse order, for backward spans. Forward span always preceded the corresponding backward span but order of presentation of digit and letter span tests was balanced across participants. The span tests were administered in the beginning of a test session including other cognitive tests.

### RESULTS

For the WM task there was no significant difference in performance between groups on either span size, *t*_(52)_ = 0.55, *p* = 0.58, *r* = 0.07, or PCS, *t*_(52)_ = 0.14, *p* = 0.89, *r* = 0.02, as in Experiment 1 and in line with our prediction (**Table 1**). Again, there was no difference in performance between groups in the manipulation phase, *t*_(51)_ = 0.31, *p* = 0.76, *r* = 0.04 (data from one participant missing due to technical problems).

Analysis of the STM span experiment showed no significant main effect of group, *F*_(1,52)_ = 0.53, *p* = 0.47, *partial* η^2^ = 0*.*01. Just as in Experiment 1, digit span was longer than letter span, *F*_(1,52)_ = 12.57, *p* = 0.001, *partial* η^2^= 0*.*20 (see **Figure 2**). There was no significant interaction effect between groups and character, *F*_(1,52)_ = 0.288, *p* = 0.594, *r* = 0.074. However, because we predicted shorter digit but not letter spans for deaf signers than hearing non-signers, we investigated and found that there was no effect of group for either digit span, *F*_(1,52)_ = 0.79, *p* = 0.38, *partial* η^2^ = 0*.*02 or letter span *F*_(1,52)_ = 0.22, *p* = 0.64, *partial* η^2^ = 0*.*004. As expected, forward spans were significantly longer than backward spans, *F*_(1,52)_ = 10.64, *p* = 0.002, *partial* η^2^ = 0.17. Opposite to our prediction of shorter backward than forward spans for hearing non-signers but not deaf signers we found longer forward than backward spans in BDS, *F*_(1,23)_ = 8.58, *p* = 0.01, *partial* η^2^ = 0.27, but not for BHN, *F*_(1,29)_ = 2.02, *p* = 0.17, *partial* η^2^= 0.07. None of the other interactions approached statistical significance (all *p*s > 0.56).

### DISCUSSION

The pattern of results obtained from the STM experiment differed somewhat between Experiments 1 and 2. Results of Experiment 2 showed no significant difference between deaf signers and hearing non-signers on either the digit or letter span tasks. Further, forward spans were longer than backward spans in Experiment 2. This contrasts with Experiment 1 where we did not find such an effect. We reasoned that the lack of a directional effect in Experiment 1 was driven by visual presentation and keypress response, both of which are known to reduce the recency effect which contributes to the reported performance difference between recall of first compared to recall of last presented items (Beaman and Morton, 2000; Cowan et al., 2004). In Experiment 2 we used handwritten response which influences recency less (Penney and Blackwood, 1989). As expected, with handwritten response, performance was worse for backward compared to forward recall, however, in contrast to our prediction, only for deaf signers.

In the operation span task, which was our measure of WM, we found no significant differences in performance between BDS and BHN. However, it is important to note here that the performance of both groups approached ceiling. Thus, there is a possibility that the operation span task was not powerful enough to detect group differences in Experiment 2. However, in Experiment 1, there was no evidence of a ceiling effect and no difference in performance between deaf and hearing participants on WM. Both experiments formed part of larger studies reported elsewhere. In Experiment 1, participants performed the span tasks towards the end of an extensive test battery, when they were probably tired. In Experiment 2, participants performed the span tasks when they were still fresh. This may explain why performance approached ceiling in Experiment 2 but not in Experiment 1. This explanation also suggests that the lack of difference in WM performance between groups in Experiment 2 is real and not simply an artifact of a ceiling effect.

## CROSS EXPERIMENT ANALYSIS

The main aim of the cross experiment analysis was to determine whether the similarities and differences in performance across character and language modality in Experiments 1 and 2 would crystalize into a more general pattern of results. A further aim was to determine whether an interaction between response direction and language modality would become apparent with the greater power afforded by collapsing the data.

### MATERIALS AND METHODS

The data entered into the cross experiment analysis were identical to those analyzed in Experiments 1 and 2. As deaf signer and hearing non-signer groups with similar characteristics took part in both experiments, we set up a design with two between-group factors for the STM tasks. The first factor, also analyzed in Experiment 1 and 2, was language modality. The second factor, which was new for the cross experiment analysis, was data set (response mode). There was an age difference between data sets such that British participants (BDS and BHN) were significantly older than the Swedish participants (SDS and SHN: *F*_(1,86)_ = 15.2, *p* < 0.001, *r* = 0.39). However, no age difference between deaf signers and hearing non-signers, *F*_(1,86)_ = 0.84, *p* = 0.36, *r* = 0.09, were found. Performance on the non-verbal intelligence tests could not be compared since Raven’s SPM (Experiment 1) does not differentiate as well as WASI (Experiment 2) in the higher ranges. However, all participants performed within the normal range on the non-verbal IQ scale.

#### Statistics

For the cross experiment analysis of the STM tasks the ANOVA design was 2 × 2 × 2 × 2: language modality (deaf signers, hearing non-signers) × response mode (key press, handwritten) × character (digits, letters) × direction of recall (forward, backward). There were two between-subject factors: modality (deaf signers and hearing non-signers) and response mode (key press and written) and the within-subject factors were, as in Experiments 1 and 2, direction of recall (forward and backward) and character (digit and letter).

### RESULTS

The British participants had significantly longer STM spans than the Swedish participants, *F*_(1,86)_ = 11.74, *p* = 0.001, *partial* η^2^ = 0.12. Further, there was a non-significant tendency towards shorter spans for deaf signers than hearing non-signers, *F*_(1,86)_ = 2.41, *p* = 0.12, *partial* η^2^ = 0*.*03. Digit span was longer than letter span, *F*_(1,86)_ = 33.47, *p* < 0.001, *partial* η^2^ = 0.28. Testing the hypothesis that deaf signers had shorter digit but not letter spans than hearing non-signers, as in Experiment 1, we investigated the non-significant interaction between character and language modality, *F*_(1,86)_ = 1.69, *p* = 0.197, *partial* η^2^ = 0.02 and found an effect of shorter digit spans for deaf signers than hearing non-signers, *F*_(1,88)_ = 3.96, *p* = 0.05, *partial* η^2^ = 0*.*04, but no difference in letter span length between language modalities, *F*_(1,88)_ = 0.98, *p* = 0.33, *partial* η^2^ = 0.01. Forward spans were longer than backward spans, *F*_(1,86)_ = 11.05, *p* = 0.001, *partial* η^2^ = 0*.*11. Because we had predicted shorter backward than forward spans for hearing non-signers but not deaf signers, the non-significant interaction between language modality and direction, *F*_(1,86)_ = 0.491, *p* = 0.485, *partial* η^2^ = 0.01, was tested and we found longer forward than backward spans for both deaf signers, *F*_(1,41)_ = 7.12, *p* = 0.01, *partial* η^2^ = 0.15, and hearing non-signers, *F*_(1,47)_ = 4.58, *p* = 0.04, *partial* η^2^ = 0.09.

To exclude the possibility that age drove the difference in STM performance between the British and the Swedish groups, we re-ran the analysis, including age as a covariate. However, the Swedish participants still performed worse than the British participants, *F*_(1,85)_ = 15.45, *p* < 0.001, *partial* η^2^ = 0.15 and the effect of character, *F*_(1,85)_ = 4.01, *p* = 0.049, *partial* η^2^ = 0.05, and direction, *F*_(1,85)_ = 6.78, *p* = 0.01, *partial* η^2^ = 0.07, still remained. To exclude the possibility that the absence of between-group differences in WM performance in Experiments 1 and 2 was due to lack of power, we compared deaf signing and hearing non-signing groups across experiments but still found no difference for either span size, *t*_(87)_ = 0.31, *p* = 0.76, *r* = 0.03, or PCS, *t*_(87)_ = 0.83, *p* = 0.41, *r* = 0.09.

### DISCUSSION

The cross experiment analysis generated three important results. Firstly, the lack of differences between groups on WM persisted. Secondly, a clearer pattern crystalized concerning the effects of character and language modality in STM. In particular, the pattern of results revealed in Experiment 1 was confirmed and strengthened. Hearing non-signers had longer digit spans than deaf signers, while there was no difference in letter span length. This is in line with our hypothesis that the formational similarity of manual numerals representing digits causes a phonological similarity effect leading to poorer STM performance. Thirdly, despite the increase in power achieved by collapsing over data sets there was still no difference in the effect of direction for signers and speakers.

Further, we found that the British participants performed better than the Swedish participants. We have already noted that British participants performed span testing while they were still fresh whereas Swedish participants had been subject to an extensive battery of testing when they performed the tasks included in the present study. Thus, one likely explanation of performance difference across experiments is a fatigue effect. The participants of the four groups included in the cross experiment analysis all had normal non-verbal intelligence and had completed at least mandatory education in their respective country. Further, they were similar on language proficiency. The age difference did not affect the results materially. Even though we cannot rule out that there are differences in non-verbal intelligence and in level of education which we have not been able to control for, that could affect the results, we do not believe that this is the main cause of the difference in performance between experiments. Of course there was one important difference in the design of the two experiments, namely response mode. Thus, although we believe a fatigue effect may be driving the differences in performance between the two experiments, we cannot rule out an effect of response mode.

## EXPERIMENT 3

To determine whether the difference in STM performance between the Swedish and British populations could be attributed to difference in response mode, we performed a third experiment with normally hearing Swedish participants who performed forward and backward versions of the digit and letter span tasks with both key press and handwritten response modes in a fully within-subjects design.

Short-term memory span size has been found to be approximately equivalent to the number of items that can be articulated in 2 s (Baddeley et al., 1975). Speakers of languages with short digit names, such as Chinese, show longer digit spans than speakers of languages with longer digit names, such as Welsh (Ellis and Hennelly, 1980; Elliott, 1992). To investigate if the longer digit than letter span found in Experiment 1 and 2 could be attributed to slower articulation rate for letters, we also determined relative articulation rates for the digits and letters used in the lists.

### MATERIALS AND METHODS

#### Participants

Sixteen adults (eight women; mean age = 32.63, *SD* = 5.46) with normal hearing took part in the experiment. All participants had at least 3 years of university education. Written informed consent was given by all participants. No compensation was paid.

#### Experimental design

The stimuli from Experiment 1 were used along with an additional set of material generated according to the principles described in Experiment 1. Specifically the four lists of digit sequences (two old and two new) and the four lists of letter sequences (two old and two new) were randomized across the four different conditions (forward and backward by keypress and by handwritten response). Power calculation showed that 16 participants would be sufficient to reveal an effect equivalent to that found between British and Swedish participants. Taking practical considerations into account, we decided to let forward span always precede the equivalent backward span in the same response mode but to switch response mode between these span pairs. Character and response mode order were balanced. This resulted in 16 different test order lists and participants were randomized to these lists. The participants were also given separate lists of 200 digits and letters and asked to say them aloud as fast as possible (based on Boutla et al., 2004). Time was taken and rounded to the nearest whole second.

#### Statistics

For the STM tasks the ANOVA design was 2 × 2 × 2: response mode (key press, handwritten) × character (digits, letters) × direction of recall (forward, backward). The articulation rate measures were analyzed by a *t*-test and further run together with the STM data in a correlation analysis.

### RESULTS

**FIGURE 3:**
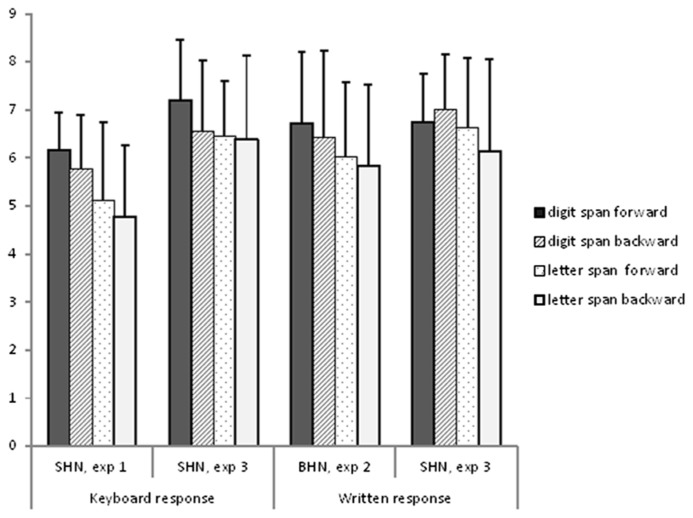
**Mean forward and backward digit and letter span for all hearing non-signers from Experiment 1 to 3 divided by response mode and cultural setting**.

The results of the span tasks are shown together with corresponding results from Experiment 1 and 2 in **Figure 3**. As in Experiments 1 and 2, digit span was longer than letter span, *F*_(1,15)_ = 6.99, *p* = 0.02, *partial* η^2^ = 0*.*32. However, there was no difference in direction, *F*_(1,15)_ = 0.82, *p* = 0.381, *partial* η^2^ = 0*.*05,** or response mode, *F*_(1,15)_ = 0.01, *p* = 0.93, *partial* η^2^ = 0.001 and none of the two-way interactions approached statistical significance (all *p*s > 0.4).

The set of 200 digits were articulated significantly more rapidly (*M* = 69.63, *SD* = 12.85) than the set of 200 letters (*M* = 76.81, *SD* = 9.70), *t*_(15)_ = 2.71, *p* = 0.02, *r* = 0.57. However, there were no significant correlations between the span scores and articulation rate (all *p*s > 0.28). Thus, the better performance in digit span than letter span cannot be explained by a slower articulation rate for letters than digits.

To test if the lack of a directional effect in Experiment 1 and 3 is due to lack of power we collapsed the data from all hearing participants in Experiment 1 and 3, but were still not able to find an effect of direction, *F*_(1,33)_ = 1.92, *p* = 0.18, *partial* η^2^ = 0.06.

Finally, we investigated if there were differences between keyboard response in Experiment 3 and SHN from Experiment 1 and between written response in Experiment 3 and BHN from Experiment 2. We found that the non-signers in Experiment 3 had a significantly better overall performance than non-signers in Experiment 1, *F*_(1,32)_ = 14.02, *p* = 0.001, *partial* η^2^** = 0.31, whereas there were no difference between the non-signers in Experiment 2 and 3, *F*_(1,44)_ = 0.88, *p* = 0.35, *partial* η^2^** = 0.02.

### DISCUSSION

The results of Experiment 3 demonstrated that the difference in STM span performance between the Swedish and British participants observed in the cross experiment analysis cannot be attributed to the use of different response modes. Because the participants enrolled in Experiment 3 performed on par with the non-signers in Experiment 2 and significantly better than the non-signers in Experiment 1 the significant difference in performance between the Swedish and British participants found in the cross experiment analysis is probably due to differences in the overall design of the studies in which the individual experiments were included and lack of participant matching between experiments. In particular, the educational background of the participants differed between Experiments 1 and 3. Whereas all participants in Experiment 3 had at least 3 years of university education, only one-third of the participants in each of the two groups in Experiment 1 had a university degree. Level of education is positively correlated with cognition (e.g., Kuncel et al., 2004; St Clair-Thompson and Gathercole, 2006), and thus it is plausible that better STM performance in Experiment 3 compared to Experiment 1 was due to differences in level of education (Unsworth and Engle, 2007).

The lack of directional effect mirrors the results of Experiments 1 and 2 for non-signing participants. As we have already noted, the visual presentation used in the present study is likely to reduce the effect of recall direction. Interestingly, several participants commented after completing the testing that they were surprised to find backward recall easier than forward recall.

We found faster articulation rates for digits than letters but we found no association between articulation rate and span size for either digits nor letters. Thus, although we cannot exclude the possibility that articulation rate explains some of the between-group differences in STM performance, the evidence suggests that it does not constitute a key underlying mechanism. This contrasts to other studies where such a correlation has been described for both digit and letter span in hearing individuals (Hall and Bavelier, 2010).

## GENERAL DISCUSSION

In the present study, we investigated WM and STM using a novel approach based on keeping stimulus presentation and response modes constant across groups, while assuming similar recoding demands in relation to memory encoding and recall across groups. For the first time, we investigated WM performance between well-matched groups of deaf signers and hearing non-signers on a digit-based operation span task and were unable to identify differences in performance. In line with the literature, we also showed poorer STM performance for deaf signers compared to hearing non-signers on a digit span task. This suggests that deaf signers and hearing non-signers have equivalent digit-based WM despite poorer STM performance for deaf signers compared to hearing non-signers on a digit span task. These findings confirm and extend previous work demonstrating similar WM performance for deaf signers and hearing non-signers on a production span task involving storage and processing of lexical items (Boutla et al., 2004). This suggests that the WM processing capacity of deaf signers can outweigh any STM storage decrement, even when WM processing involves digits.

In the present study we used operation span as a measure of WM and found no differences between deaf signers and hearing non-signers. This well-established test loads strongly on overall WM capacity (Unsworth and Engle, 2005, 2007). It also has the advantage of being digit-based, thus making it comparable with the digit span test of STM and avoiding the use of speech-based lexical items which are known to make different cognitive processing demands on deaf signers and hearing non-signers. The mechanisms behind similar WM but different STM for deaf signers and hearing non-signers on digit-based tasks need to be investigated experimentally and it may turn out that the storage component is more heavily taxed for deaf signers in both tasks. However, one explanation may be a different or more efficient allocation of resources between the storage and processing components of WM for deaf signers than hearing non-signers, which would compensate for poorer STM storage for digits. Such an effect may be related to the ability of signers to generate propositions at the same rate as speakers with economical use of lexical items (Bellugi and Fischer, 1972). One of the main functions of WM is the comprehension and generation of propositions (Baddeley, 2003).

For the first time we present results from a cross cultural analysis of STM in deaf signers and hearing non-signers. There was a difference in performance between Experiments 1 and 2 with British participants showing consistently better STM performance than Swedish participants. Experiment 3 showed that better performance by British than Swedish participants could not be explained by the difference in response mode between Experiments 1 and 2. Instead, we suggest that the reason for the difference between experiments is twofold: Firstly, it may stem from potential differences in participant characteristics between experiments. Although these were rigorously controlled within experiments, the same was not possible between experiments. In particular, we suggest that potential differences in level of education between experiments may have driven the differences in STM. Secondly, as already suggested participants in Experiment 1 may have been more fatigued than participants in Experiment 2 and 3 when they performed the span tasks.

Experiment 1 provided some evidence of STM performance differences relating to language modality (sign and speech) and character (digit and letter) in support of our hypothesis. In Experiment 1 and in the cross experiment analysis, we found evidence of shorter digit but not letter spans for sign than speech for the first time in one and the same analysis. Shorter digit span for deaf signers compared to hearing non-signers was shown previously by Bavelier et al. (2008) and no difference between deaf signers and hearing non-signers on letter span was shown by Wilson and Emmorey (2006a). The results of the present study are in line with our hypothesis, based on Wilson and Emmorey (1997a, 2006b), that the formational similarity of manual numerals representing digits, compared to the phonological distinctiveness of spoken digits, would generate a phonological similarity effect leading to poorer STM performance for signers than speakers, whereas no such effect would be apparent for letters chosen explicitly to minimize the formational similarity of their sign names and the phonological similarity of their spoken names.

There was no evidence of shorter letter spans for DS than HN as would have been predicted by the findings of Bavelier et al. (2008). At least three factors differentiate the present study from the study by Bavelier et al. (2008) where group differences were found for both digit and letter span. The first is that the present study used a within-group comparison of digit and letter span, guarding against sampling differences leading to a more stringent comparison between the two span types. The second is that groups in the present study were matched on non-verbal intelligence, whereas any cognitive differences between the groups may have confounded the observed effects in Bavelier et al. (2008). The third and presumably the most important difference, relates to presentation mode, where the present study makes consistent use of text-based visual presentation, whereas Bavelier et al. (2008) used auditory presentation for hearing non-signers and signed presentation for deaf signers. For both deaf signers and hearing non-signers text-based visual presentation probably requires phonological recoding. However, because visual presentation removes the opportunity for the hearing individuals to take advantage of longer lasting auditory memory traces (Cowan, 2000), it is expected that the performance of the hearing individuals decreases more than that of the deaf individuals in the visual presentation mode. Therefore, we suggest that the visual presentation employed in the present study makes group comparisons more equal and that the remaining difference between groups on the digit span task reflects the “true” difference between groups and that this difference is related to differences in the phonological similarity of recoded digits between the two language modalities of sign and speech. However, because the language modality by character interactions were not statistically significant, possibly due to insufficient power, simple main effects should be interpreted with caution.

The lack of digit span difference between deaf signers and hearing non-signers in Experiment 2 was unexpected but might indicate an element of speech phonology in the STM representation of printed digits by BDS (Hanson, 1982). All but one of the SDS had been educated mainly in SSL in accordance with the 1983 Swedish National school curriculum (Svartholm, 2010). No comparable national curriculum exists in Britain and individual schools have varying communication policies. Thus, the BDS group was less likely to have had a strong emphasis on sign language during their education and might be expected to use speech phonology in representation of printed digits and letters to a greater extent than SDS and might therefore not be affected to the same extent by the phonological similarity of the manual numerals.

We predicted that if temporal processing differences between signed and spoken languages influence STM, hearing non-signers – but not deaf signers – would perform worse on backward than forward recall. We did not find any evidence supporting this in either Experiment 1 or 2 or in the cross experimental analysis. This is in line with Gozzi et al. (2011) who found no support for serial order being a detrimental factor for the STM discrepancy between signers and speakers. Some of the work on which we based our hypothesis studied STM in children (e.g., O’Connor and Hermelin, 1973; Wilson et al., 1997). Deficits in the allocation of attention in time sometimes found in deaf children largely resolve by adulthood (Dye and Bavelier, 2010). Thus, it is possible that lower STM in deaf children is partially explained by deficits in temporal processing not found in deaf adults. However, other recent studies have shown differences in temporal order processing in adult signers and speakers in STM using free recall (Bavelier et al., 2008) and WM using temporal versus spatial modes of presentation (Rudner et al., 2010). The temporal processing manipulation in the present study involved direction of recall. Both forward and backward recall require maintenance of temporal order while processing differs between directions. In line with Bavelier et al. (2008), we conclude that the relative difference in temporal processing demands between forward and backward serial recall does not differ between the language modalities of sign and speech in adult deaf signers and hearing non-signers.

## CONCLUSION

For the first time we have shown similar *digit-based* WM performance for deaf signers and hearing non-signers in both Swedish and British populations. This extends previous findings of similar lexically based WM for signers and non-signers. Importantly, this shows that deaf signers and hearing non-signers can have equivalent digit-based WM despite poorer digit-based STM. Further, we have shown that poor digit span performance for deaf signers compared to hearing non-signers is probably due to the greater phonological similarity for deaf signers, since no between-group differences were found for letter span. We found no differences between deaf signers and hearing non-signers in the relative effect of recall direction, suggesting that these particular temporal processing demands do not play out differently in STM for these two groups when stimuli are printed. Nonetheless, because simple span tests seem to be confounded by phonological similarity, we suggest that WM tasks, either verbal or digit-based, may provide a better test of cognitive function in deaf individuals.

## Conflict of Interest Statement

The authors declare that the research was conducted in the absence of any commercial or financial relationships that could be construed as a potential conflict of interest.

## AUTHOR CONTRIBUTIONS

Experiment 1 was prepared and designed by Josefine Andin, Jerker Rönnberg, and Mary Rudner, Experiment 2 by Josefine Andin, Jerker Rönnberg, Mary Rudner, Eleni Orfanidou, Cheryl M. Capek and Bencie Woll and Experiment 3 by Josefine Andin, Mary Rudner, and Emil Holmer. Acquisition of data was done by Josefine Andin together with interpreter Lena Davidsson (acknowledged) in Experiment 1, by Eleni Orfanidou, Velia Cardin, and Sally Reynolds (acknowledged) in Experiment 2 and by Emil Holmer in Experiment 3. Analysis and interpretation of results was carried out mainly by Josefine Andin and Mary Rudner in Experiment 1 and Experiment 2 and by Mary Rudner and Emil Holmer in Experiment 3. The first draft of the manuscript was written by Josefine Andin and Mary Rudner. All authors took part in critical revision of the manuscript.

## References

[B1] Alamargot D. Lambert E. Thebault C. Dansac C. (2007). Text composition by deaf and hearing middle-school students: the role of working memory. *Read. Writ.* 20 333–360 10.1007/s11145-006-9033-y

[B2] Baddeley A. (2003). Working memory and language: an overview. *J. Commun. Disord.* 36 189–208 10.1016/S0021-9924(03)00019-4 12742667

[B3] Baddeley A. Thomson N. Buchanan M. (1975). Word length and structure of short-term memory. *J. Verbal Learn. Verbal Behav.* 14 575–589 10.1016/S0022-5371(75)80045-4

[B4] Bavelier D. Newport E. L. Hall M. Supalla T. Boutla M. (2006). Persistent difference in short-term memory span between sign and speech: implications for cross-linguistic comparisons. *Psychol. Sci.* 17 1090–1092 10.1111/j.1467-9280.2006.01831.x 17201792

[B5] Bavelier D. Newport E. L. Hall M. Supalla T. Boutla M. (2008). Ordered short-term memory differs in signers and speakers: implications for models of short-term memory. *Cognition* 107 433–459 10.1016/j.cognition.2007.10.012 18083155 PMC2396490

[B6] Beaman C. Morton J. (2000). The separate but related origins of the recency effect and the modality effect in free recall. *Cognition* 77 B59–B65 10.1016/S0010-0277(00)00107-4 11018512

[B7] Bellugi B. Fischer S. (1972). A comparison of sign language and spoken language. *Cognition* 1 173–200

[B8] Bergman B. Wikström, L.-Å (1981). Supplement till Forskning om Teckenspråk, videogram I: Svenska handalfabetet och bokstaverade tecken [Supplement to Research on sign language, videogram I; Swedish manual alphabet and fingerspelled signs]. Stockholm: Stockholms universitet

[B9] Boutla M. Supalla T. Newport E. L. Bavelier D. (2004). Short-term memory span: insights from sign language. *Nat. Neurosci.* 7 997–1002 10.1038/nn1298 15311279 PMC2945821

[B10] Brentari D. (1998). *A Prosodic Model of Sign Language Phonology*. Cambridge, MA: MIT Press

[B11] Carmel S. J. (1982). *International Hand Alphabet Charts*. Silver Spring, MD: National Association of the Deaf

[B12] Conrad R. (1970). Short-term memory processes in the deaf. *Br. J. Psychol.* 61 179–195 5487452 10.1111/j.2044-8295.1970.tb01236.x

[B13] Conrad R. Hull A. (1968). Input modality and the serial position curve in short-term memory. *Psychon. Sci.* 10 135–136

[B14] Cowan N. (2000). The magical number 4 in short-term memory: a reconsideration of mental storage capacity. *Behav. Brain Sci.* 24 87–185 10.1017/S0140525X01003922 11515286

[B15] Cowan N. Saults J. Brown G. (2004). On the auditory modality superiority effect in serial recall: separating input and output factors. *J. Exp. Psychol. Learn. Mem. Cogn.* 30 639–644 10.1037/0278-7393.30.3.639 15099132

[B16] Darwin C. J. Turvey M. T. Crowder R. G. (1972). An auditory analogue of the Sperling partial report procedure: evidence for brief auditory storage. *Cogn. Psychol.* 3 255–267 10.1016/0010-0285(72)90007-2

[B17] Dye M. W. G. Bavelier D. (2010). Differential development of visual attention skills in school-age children. *Vision Res.* 50 452–459 10.1016/j.visres.2009.10.010 19836409 PMC2824025

[B18] Elliott J. M. (1992). Forward digit span and articulation speed for Malay, English, and 2 Chinese dialects. *Percept. Mot. Skills* 74 291–295 10.2466/pms.1992.74.1.291

[B19] Ellis N. C. Hennelly R. A. (1980). A bilingual word-length effect: implications for intelligence testing and the relative ease of mental calculation in Welsh and English. *Br. J. Psychol.* 71 43–51 10.1111/j.2044-8295.1980.tb02728.x

[B20] Emmorey K. (2002). *Language, Cognition and the Brain: Insights from Sign Language Research*. Mahwah, NJ: Lawrence Erlbaum Associates

[B21] Forster K. Forster J. (2003). A windows display program with millisecond accuracy. *Behav. Res. Methods Instrum. Comp.* 35 116–124 10.3758/BF03195503 12723786

[B22] Geraci C. Gozzi M. Papagno C. Cecchetto C. (2008). How grammar can cope with limited short-term memory: simultaneity and seriality in sign languages. *Cognition* 106 780–804 10.1016/j.cognition.2007.04.014 17537417

[B23] Gozzi M. Geraci C. Cecchetto C. Perugini M. Papagno C. (2011). Looking for an explanation for the low sign span. Is order involved? *J. Deaf Stud. Deaf Educ.* 16 101–107 10.1093/deafed/enq035 20679138

[B24] Hall M. Bavelier D. (2010). “Working memory, deafness, and sign language,” in *Oxford Handbook of Deaf Studies, Language, and Education*, Vol.2 edsMarschark M. Spencer P. A. (London: Oxford University Press) 458–472

[B25] Hall M. Bavelier D. (2011). Short-term memory stages in sign vs. speech: the source of the serial span discrepancy. *Cognition* 120 54–66 10.1016/j.cognition.2011.02.014 PMC3095773 21450284

[B26] Hanson V. L. (1982). Short-term recall by deaf signers of American sign language: implications of encoding strategy for order recall. *J. Exp. Psychol. Learn. Mem. Cogn.* 8 572–583 10.1037/0278-7393.8.6.572 6218222

[B27] Harvey A. Beaman C. (2007). Input and output modality effects in immediate serial recall. *Memory* 15 693–700 10.1080/09658210701644677 17924278

[B28] Klima E. S. Bellugi U. (1976). Poetry and song in a language without sound. *Cognition* 4 45–97 10.1016/0010-0277(76)90010-x

[B29] Klima E. S. Bellugi U. (1979). *The Signs of Language*. Cambridge, MA: Harvard University Press

[B30] Koo D. Crain K. LaSasso C. Eden G. F. (2008). Phonological awareness and short-term memory in hearing and deaf individuals of different communication backgrounds. *Learn. Skill Acquist. Read. Dyslexia* 1145 83–99 10.1196/annals.1416.025 19076391

[B31] Kuncel N. R. Hezlett S. A. Ones D. S. (2004). Academic performance, career potential, creativity, and job performance: can one construct predict them all? *J.Pers. Soc. Psychol.* 86 148–161 10.1037/0022-3514.86.1.148 14717633

[B32] Li S. C. Lewandowsky S. (1995). Forward and backward recall: different retrieval-processes. *J. Exp. Psychol. Learn. Mem. Cogn.* 21 837–847 10.1037/0278-7393.21.4.837

[B33] Locke J. L. Locke V. L. (1971). Deaf children’s phonetic, visual and dactylic coding in a grapheme recall task. *J. Exp. Psychol.* 89 142–146 5569619 10.1037/h0031226

[B34] Marschark M. Mayer T. S. (1998). Interactions of language and memory in deaf children and adults. *Scand. J. Psychol.* 39 145–148 10.1111/1467-9450.393069 9800528

[B35] O’Connor N. Hermelin B. (1973). Short-term memory for the order of pictures and syllables by deaf and hearing children. *Neuropsychologia* 11 437–442 10.1016/0028-3932(73)90031-6 4758187

[B36] Penney C. G. Blackwood P. A. (1989). Recall mode and recency in immediate serical recall: computer users beware. *Bull. Psychon. Soc.* 27 545–547

[B37] Pintner R. Paterson D. G. (1917). A comparison of deaf and hearing children in visual memory for digits. *J. Exp. Psychol.* 2 76–88 10.1037/h0072870

[B38] Rönnberg J. Archer T. Ohlsson K. (1980). Temporal factors in audition and vision: a functional emphasis. *Scand. J. Psychol.* 21 241–247 10.1111/j.1467-9450.1980.tb00367.x 7268320

[B39] Rönnberg J. Nilsson L. G. (1987). The modality effect, sensory handicap, and compensatory functions. *Acta Psychol.* 65 263–283 10.1016/0001-6918(87)90053-9

[B40] Rönnberg J. Rudner M. Ingvar M. (2004). Neural correlates of working memory for sign language. *Cogn. Brain Res.* 20 165–182 10.1016/j.cogbrainres.2004.03.002 15183389

[B41] Rosen V. M. Engle R. W. (1997). Forward and backward serial recall. *Intelligence* 25 37–47 10.1016/S0160-2896(97)90006-4

[B42] Ross B. M. (1969). Sequential visual memory and the limited magic of number seven. *J. Exp. Psychol.* 80 339–347 4182333 10.1037/h0027274

[B43] Rudner M. Davidsson L Rönnberg J. (2010). Effects of age on the temporal organization of working memory in deaf signers. *Aging Neuropsychol. Cogn.* 17 360–383 10.1080/13825580903311832 19921581

[B44] Rudner M. Fransson P. Ingvar M. Nyberg L Rönnberg J. (2007). Neural representation of binding lexical signs and words in the episodic buffer of working memory. *Neuropsychologia* 45 2258–2276 10.1016/j.neuropsychologia.2007.02.017 17403529

[B45] Rudner M. Karlsson T. Gunnarsson J Rönnberg J. (2013). Levels of processing and language modality specificity in working memory. *Neuropsychologia* 51 656–666 10.1016/j.neuropsychologia.2012.12.011 23287569

[B46] Rudner M Rönnberg J. (2008). Explicit processing demands reveal language modality-specific organization of working memory. *J. Deaf Stud. Deaf Educ.* 13 466–484 10.1093/deafed/enn005 18353759 PMC2533441

[B47] Sandler W. Lillo-Martin D. (2006). *Sign Language and Linguistic Universals*. Cambridge, NY: Cambridge University Press

[B48] Shand M. A. Klima E. S. (1981). Nonauditory suffix effects in congenitally deaf signers of American sign language. *J. Exp. Psychol. Hum. Learn. Mem.* 7 464–474 10.1037/0278-7393.7.6.464 7328396

[B49] Smyth M. M. Hay D. C. Hitch G. J. Horton N. J. (2005). Serial position memory in the visual-spatial domain: reconstructing sequences of unfamiliar faces. *Q. J. Exp. Psychol. A Hum. Exp. Psychol.* 58 909–930 10.1080/02724980443000412 16194941

[B50] Sperling G. (1960). The information available in brief visual presentation. *Cogn. Psychol.* 74 1–29

[B51] St Clair-Thompson H. L. Gathercole S. E. (2006). Executive functions and achievements in school: shifting, updating, inhibition, and working memory. *Q. J. Exp. Psychol.* 59 745–759 10.1080/17470210500162854 16707360

[B52] Sutton-Spence R. Woll B. (1999). *The Linguistics of British Sign Language*. Cambridge: Cambridge University Press

[B53] Svartholm K. (2010). Bilingual education for deaf children in Sweden. *Int. J. Biling. Educ. Biling.* 13 159–174 10.1080/13670050903474077

[B54] Traxler C. B. (2000). The Stanford Achievement Test, 9th Edition: National Norming and Performance Standards for Deaf and Hard-of-Hearing Students. *J. Deaf Stud. Deaf Educ.* 5 337–348 10.1093/deafed/5.4.337 15454499

[B55] Turner M. L. Engle R. W. (1989). Is working memory capacity task dependent? *J. Mem. Lang.* 28 127–154 10.1016/0749-596X(89)90040-5

[B56] Unsworth N. Engle R. W. (2005). Working memory capacity and fluid abilities: examining the correlation between operation span and raven. *Intelligence* 33 67–81 10.1016/j.intell.2004.08.003

[B57] Unsworth N. Engle R. W. (2007). On the division of short-term and working memory: an examination of simple and complex span and their relation to higher order abilities. *Psychol. Bull.* 133 1038–1066 10.1037/0033-2909.133.6.1038 17967093

[B58] Wallace G. Corballis M. C. (1973). Short-term memory and coding strategies in the deaf. *J. Exp. Psychol.* 99 334–348 4742547 10.1037/h0035372

[B59] Wilson M. Bettger J. Niculae I. Klima E. (1997). Modality of language shapes working memory: evidence from digit span and spatial span in ASL signers. *J. Deaf Stud. Deaf Educ.* 2 150–160 10.1093/oxfordjournals.deafed.a014321 15579844

[B60] Wilson M. Emmorey K. (1997a). A visuospatial “phonological loop” in working memory: evidence from American sign language. *Mem. Cogn.* 25 313–320 10.3758/BF03211287 9184483

[B61] Wilson M. Emmorey K. (1997b). Working memory for sign language: a window into the architecture of the working memory system. *J. Deaf Stud. Deaf Educ.* 2 121–130 15579841 10.1093/oxfordjournals.deafed.a014318

[B62] Wilson M. Emmorey K. (1998). A “word length effect” for sign language: further evidence for the role of language in structuring working memory. *Mem. Cogn.* 26 584–590 10.3758/BF03201164 9610126

[B63] Wilson M. Emmorey K. (2006a). Comparing sign language and speech reveals a universal limit on short-term memory capacity. *Psychol. Sci.* 17 682–683 10.1111/j.1467-9280.2006.01766.x 16913950

[B64] Wilson M. Emmorey K. (2006b). No difference in short-term memory span between sign and speech. *Psychol. Sci.* 17 1093–1094 10.1111/j.1467-9280.2006.01835.x 17201793

